# Correlative optical photothermal infrared and X-ray fluorescence for chemical imaging of trace elements and relevant molecular structures directly in neurons

**DOI:** 10.1038/s41377-021-00590-x

**Published:** 2021-07-22

**Authors:** Nadja Gustavsson, Agnes Paulus, Isak Martinsson, Anders Engdahl, Kadda Medjoubi, Konstantin Klementiev, Andrea Somogyi, Tomas Deierborg, Ferenc Borondics, Gunnar K. Gouras, Oxana Klementieva

**Affiliations:** 1 Medical Microspectroscopy, Department of Experimental Medical Science, Lund University, 22180 Lund, Sweden; 2 Experimental Neuroinflammation Lab, Department of Experimental Medical Science, Lund University, 22180 Lund, Sweden; 3 Experimental Dementia Research, Department of Experimental Medical Science, Lund University, 22180 Lund, Sweden; 4 Synchrotron SOLEIL, L’Orme des Merisiers, 91192 Gif Sur Yvette Cedex, France; 5 MAX IV Laboratory, 22100 Lund, Sweden; 6 Lund Institute for advanced Neutron and X-ray Science (LINXS), 223 70 Lund, Sweden

**Keywords:** Optical techniques, Applied optics

## Abstract

Alzheimer’s disease (AD) is the most common cause of dementia, costing about 1% of the global economy. Failures of clinical trials targeting amyloid-β protein (Aβ), a key trigger of AD, have been explained by drug inefficiency regardless of the mechanisms of amyloid neurotoxicity, which are very difficult to address by available technologies. Here, we combine two imaging modalities that stand at opposite ends of the electromagnetic spectrum, and therefore, can be used as complementary tools to assess structural and chemical information directly in a single neuron. Combining label-free super-resolution microspectroscopy for sub-cellular imaging based on novel optical photothermal infrared (O-PTIR) and synchrotron-based X-ray fluorescence (S-XRF) nano-imaging techniques, we capture elemental distribution and fibrillary forms of amyloid-β proteins in the same neurons at an unprecedented resolution. Our results reveal that in primary AD-like neurons, iron clusters co-localize with elevated amyloid β-sheet structures and oxidized lipids. Overall, our O-PTIR/S-XRF results motivate using high-resolution multimodal microspectroscopic approaches to understand the role of molecular structures and trace elements within a single neuronal cell.

## Abstract (web summary)

A new imaging approach that combines two techniques standing at the opposite ends of the electromagnetic spectrum.

## Graphical abstract

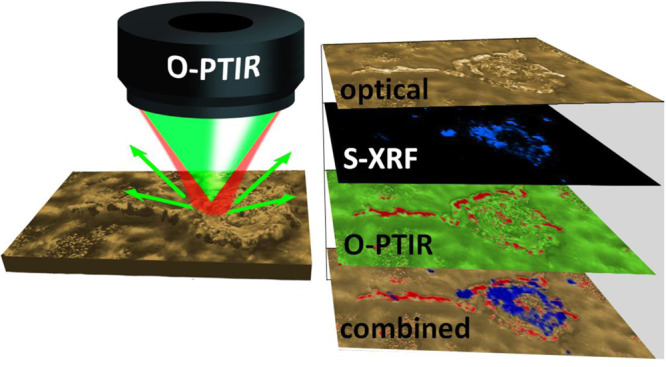

## Introduction

The amyloid hypothesis places amyloid-β protein (Aβ) as a critical trigger of Alzheimer’s disease (AD)^1^. Thus the development of many anti-amyloid drugs focuses on the reduction of Aβ concentrations. However, failures of clinical trials targeting Aβ proteins indicate that the mechanisms of AD-related neurodegeneration are more complex^2,3^. In particular, our previous work has revealed a polymorphic nature of β-sheet-structured Aβ aggregates in AD transgenic neurons, however, it is still unclear structurally polymorphic Aβ may trigger different mechanisms of neurotoxicity^4^. Therefore, to find effective anti-Aβ therapeutics against Aβ aggregation, it is critical to understand the molecular mechanisms of amyloid formation. Among all of the biological factors linked to amyloid aggregation, a cellular environment plays an important role^5^, and in particular, redox-reactive metal ions play important roles influencing amyloid plaque morphology^6–8^. As one of the possible pathological mechanisms, it has been suggested that upon binding, Aβ proteins can reduce redox-active metals and, by producing OH^•^ radicals, cause oxidative stress^9–13^. While several explanations have been proposed, molecular mechanisms of neuronal damage remain uncertain. Therefore, in situ studies of redox-active metals together with biologically relevant macromolecules such as amyloid aggregates^14^ assessed directly in a cellular environment may help us to better understand AD pathology. However, dissecting molecular mechanisms involving metal ions and structural changes of amyloid proteins in cells or tissues is a very challenging task since protein structures and metal ion concentrations can be easily affected by chemical processing. Thus, sensitive label-free imaging methods are urgently required to address protein structure and elemental distribution in cells. Infrared (IR) microscopy and X-ray fluorescence spectroscopy (XRF) have been combined to study brain tissues^7^ and brain cells^15^. However, a spatial resolution of IR/XRF approach has been determined by IR microscopy, which is diffraction-limited and strongly depends on the IR wavelength (Table S1). Typically, IR light limits traditional IR microscopy to particles >∼3 μm, which provides less relevance for resolving structures at the subcellular level.

Recent advances in nanoscale optical photothermal infrared (O-PTIR) allows one to overcome the diffraction limit of IR light, providing a spatial resolution that is no longer diffraction-limited but is determined instead by the focusing laser achieving a few hundred nanometers^16–19^. Using O-PTIR, we have demonstrated a significant increase of β-sheet content directly in AD-like neurons at a sub-cellular level^4^. In the present work, we focused on correlative label-free imaging of β-sheet content and Fe/Cu distribution in cultured primary neurons, a cellular model of AD. Specifically, to image the molecular structures, we used O-PTIR, and to analyze the composition of elements in the very same neurons, we used synchrotron-based X-ray fluorescence (S-XRF).

For this study, two aspects of O-PTIR were critical: first, the spatial resolution of O-PTIR (~300 nm)^4,20^, and second, measurements in non-contact mode. Submicron spatial resolution was necessary to trace protein structures inside cells; the non-contact mode was essential to preserve 100 nm thin Si_3_N_4_ membrane used for S-XRF imaging.

**Fig. 1:**
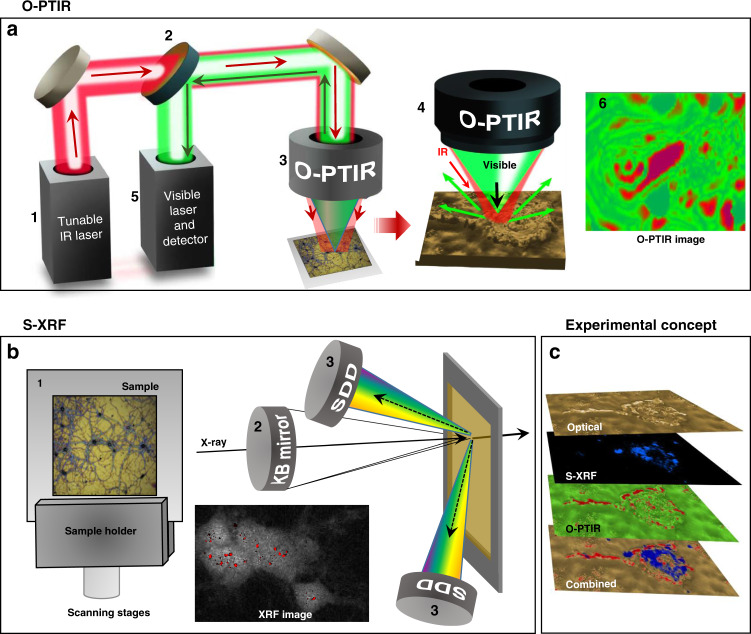
Sample preparation and experimental setup. **a** O-PTIR setup: a pulsed, tunable IR laser is guided onto the sample surface (1). The IR laser is made collinear with the green 532 nm detection laser (2). The collinear beams are focused on the sample surface through a microscope objective (3). When IR absorption occurs, the photothermal response of the sample is monitored by the scattered green light (4). The reflected green light returns to the detector, and the IR signal is extracted (5), and an infrared image is produced (6). **b** X-ray fluorescence nanoimaging of individual neuronal cells deposited on Si_3_N_4_ (1). The sample is scanned by the nanofocused beam produced by the Kirkpatrick–Baez nanofocusing mirror pair (2), while the X-ray fluorescence signal is being collected by two Si drift detectors (3). S-XRF image shows the distribution of iron at a sub-cellular level. **c** Conceptualization of the data analysis based on superimposed optical, O-PTIR, and S-XRF images

Here we present a motivating concept for single-cell structural analysis: O-PTIR/S-XRF to study amyloid aggregation processes involved by mapping molecular structures and trace metals in the same neuronal cell (Fig. 1). We explored how this interdisciplinary multimodal approach can be used to answer long-standing questions in AD, such as how metal ion dyshomeostasis can be involved in amyloid neurotoxicity. Using O-PTIR/S-XRF we demonstrated co-localization of clustered of iron with elevated amyloid β-sheet structures and oxidized lipids directly in primary cortical neurons, models of AD. Lastly, we discussed further development of the O-PTIR/S-XRF approach for cellular studies to investigate metals and amyloid structures in relation to pathological conditions promoting the formation of β-sheet structures to yield further insight into the pathogenesis of AD.

## Results

The primary goal of this proof-of-principle experiment was to combine and correlate O-PTIR and S-XRF measurements of the very same neuron and test experimental conditions that meet the O-PTIR and S-XRF requirements such as compatibility of spatial resolution and the contribution of S-XRF substrate on the O-PTIR spectra. To study a possible role of metals in amyloid-β formation, we used neurons that were grown directly on the 100 nm Si_3_N_4_ membrane (Figure S1), and after 19 days of culturing, neurons were treated with amyloid-beta protein, Aβ(1–42). When added to the cell media, Aβ(1–42) is usually absorbed by the cultured neurons within approximately 30 min^21^. To avoid artificial influences on amyloid structures, neurons were fixed in 4% paraformaldehyde and freeze-dried. Neurons were derived from the brains of mice that lack functional amyloid precursor protein (APP-KO)^22^ and therefore do not express Aβ. Thus, detected elevations of β-sheet structures in neurons were unambiguously assigned to exogenously added Aβ(1–42). To avoid changes in protein structures and metal concentrations, both easily affected by chemical processing, we used a label-free protocol that completely excludes the use of detergents or dyes. To avoid radiation damage, neurons were first examined by O-PTIR and then with S-XRF. For O-PTIR measurements, we used only 5% (~2 mW) of probe laser power, with the scan rate of 0.05 Hz for IR maps and 1 s for spectral acquisition time. Using O-PTIR, we monitored the absorbance of β-sheet and lipid oxidation. Early empirical frequency-structure studies have found that β-sheets have an absorption band between 1625 and 1640 cm^–1^ ^23,24^. The intensity of the band centered at 1740 cm^−1^ has been associated with stretching vibrations of the carbonyl group of ester bonds ν(C=O) between fatty acids and glycerol within the phospholipids molecules^25,26^ that form the backbone of neural membranes, providing fluidity and permeability^27^ and controlling synaptic neurotransmission and plasticity^28^. It has also been shown that (C=O) bonds may be formed by the peroxidation of fatty acid chains^29^. Thus, an increase in the intensity of this band indicates an increase in lipid oxidation^30^ as confirmed in the literature^25,31–37^. To visualize elevations of β-sheet structures and to select locations for spectra acquisition, ratio maps (1628/1650) were calculated for treated and control neurons as explained in Figs. S3 and S4. In the case of neurons, the IR penetration depth in O-PTIR is relative to the sample thickness^4^, therefore, to consider the neuronal thickness, we used IR maps acquired at 1650 cm^–1^ corresponding to Amide I^23^ for normalization of the IR signal.

**Fig. 2:**
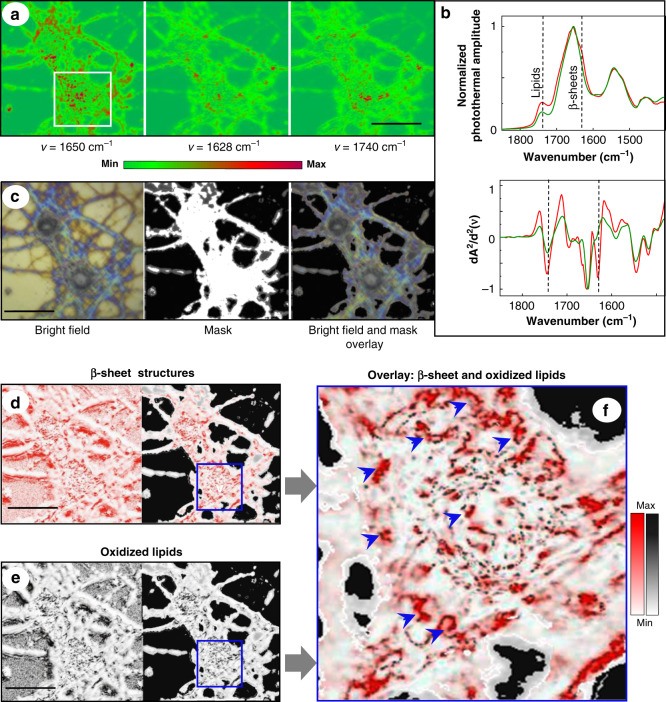
Co-localization of β-sheet structures and lipid oxidation in cultured primary neurons, a cellular model of AD. Cultured APP-KO neurons were incubated with 5 × 10^−6^ M Aβ(1–42) for 30 min. **a** O-PTIR maps acquired at frequencies 1650, 1628, and 1740 cm^–1^. Square indicates zoom-in area shown in (**f**). **b** Averaged and normalized O-PTIR spectra and their normalized second derivatives recorded from the neurons treated with Aβ (red) and untreated neurons (green). The locations for red spectra are shown in (**f**). Dashed lines indicate the band positions characteristic for β-sheet structures (1628 cm^–1^) and oxidized lipids (1740 cm^–1^). *N* = 3 cells per APP-KO embryo, from a total of 3 embryos. **c** Bright-field image of APP-KO primary neurons, image mask, and overlay of a bright-field image with the mask. **d** β-sheet map: a ratio map was derived from the image acquired at 1628 cm^–1^and divided by the image acquired at 1650 cm^–1^. **e** Lipid oxidation map: A ratio map derived from the image acquired at 1740 cm^−1^ and divided by the image acquired at 1650 cm^–1^. **f** Digital zoom-in image of the area indicated by a square in (**d**) and (**e**) as an overlay of (red dots) and oxidized lipids (gray dots). Arrows indicate example positions where spectra were taken. Colored bars show the intensity of photothermal amplitude, ranging from 0, set as a threshold, to the maximum. Scale bars are 20 µm

To visualize a co-localization of β-sheet structures and lipid oxidation, ratio maps of 1628/1650 (Fig. 2d) and 1740/1650 (Fig. 2e) were superimposed. The results showed that in Aβ-treated neurons, the spots with elevated β-sheet content are characterized by the presence of oxidized lipids (Fig. 2f). Since no elevation of β-sheet load nor lipid oxidation were observed in untreated neurons (Fig. 1b, Figs. S3 and S4), our results indicate that elevation of β-sheet load detected in diseased neurons can be associated with membrane oxidation. Importantly, for the region of interest (lipid oxidation, β-sheet folding, with the corresponding frequency between 1800 and 1500 cm^–1^), substrate contribution to the O-PTIR spectra was not detected (Fig. S2).

**Fig. 3:**
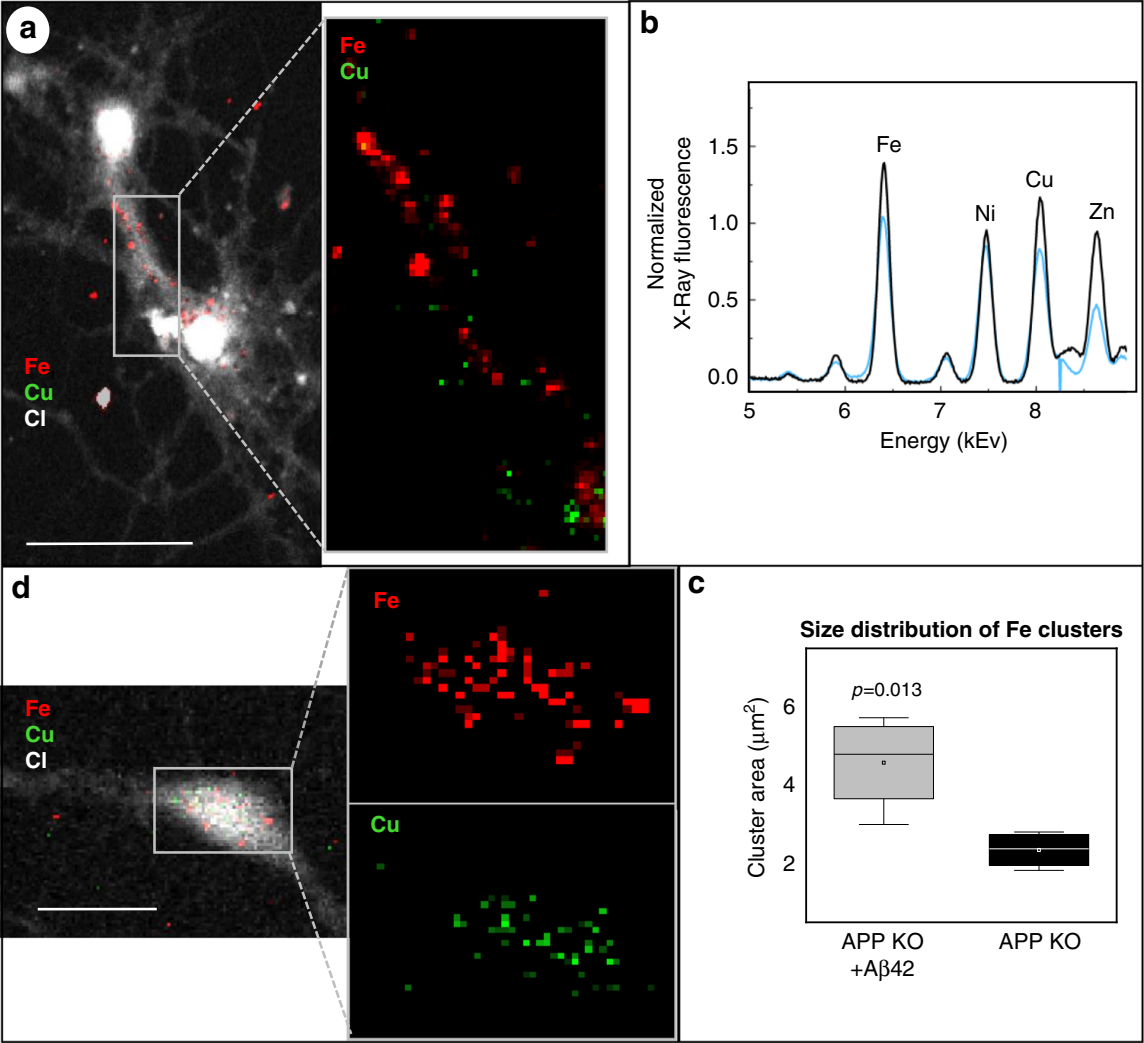
Elemental distribution imaged by scanning X-ray fluorescence nanoprobe in cultured primary neurons, a cellular model of AD. **a** Elemental distribution imaged by scanning X-ray fluorescence nanoprobe in cultured primary neurons, a cellular model of AD. **a** Elemental distribution of Cl, Cu, and Fe in cultured APP-KO neurons incubated for 30 min with 5 × 10^−6^ M synthetic Aβ(1–42). Maps of Cl are used for neuronal identification. Pixel size is 500 nm; scale bar is 40 µm. **b** Averaged and normalized S-XRF sum-spectra of cultured mouse primary neurons. Blue spectrum corresponds to APP-KO treated with Aβ(1–42); black spectrum corresponds to untreated neurons. (*N* = 3 cells per APP-KO embryo, from a total of 3 embryos). **c** Average area of the Fe clusters calculated per cell, including soma and neurites. **d** Iron distribution in untreated APP-KO primary neurons. Scale bar is 20 µm

Imaging of metal ions in primary neurons can only be performed by synchrotron-based XRF due to the need for a nano-focused high energy beam, such as that available at the Nanoscopium beamline at the synchrotron SOLEIL (France). Nanoscopium provides high spatial resolution to detect the distribution of metal ions at trace levels, in parts per million (ppm) concentration range, and has been successfully used to image metal ions in different biological samples^38–40^. Using Nanoscopium nanoprobe, we examined the elemental distribution in intact APP-KO neurons and APP-KO neurons treated with Aβ (Fig. 3a). Summative XRF spectra were normalized per image area (Fig. 3b), but not per cell area nor cell thickness. We analyzed the average size of Fe clusters by quantifying the average area of the clusters per cell, including soma and neurites. Strikingly, we observed significant Fe clustering in the treated neurons (*p* = 0.013) (Fig. 3c); such clustering of Fe was not observed in the untreated cells (Fig. 3d). Surprisingly, no significant clustering of Cu ions nor Cu concentrations as measured by inductively coupled plasma mass spectroscopy has been observed after treatment with Aβ(1–42) (data not shown).

**Fig. 4:**
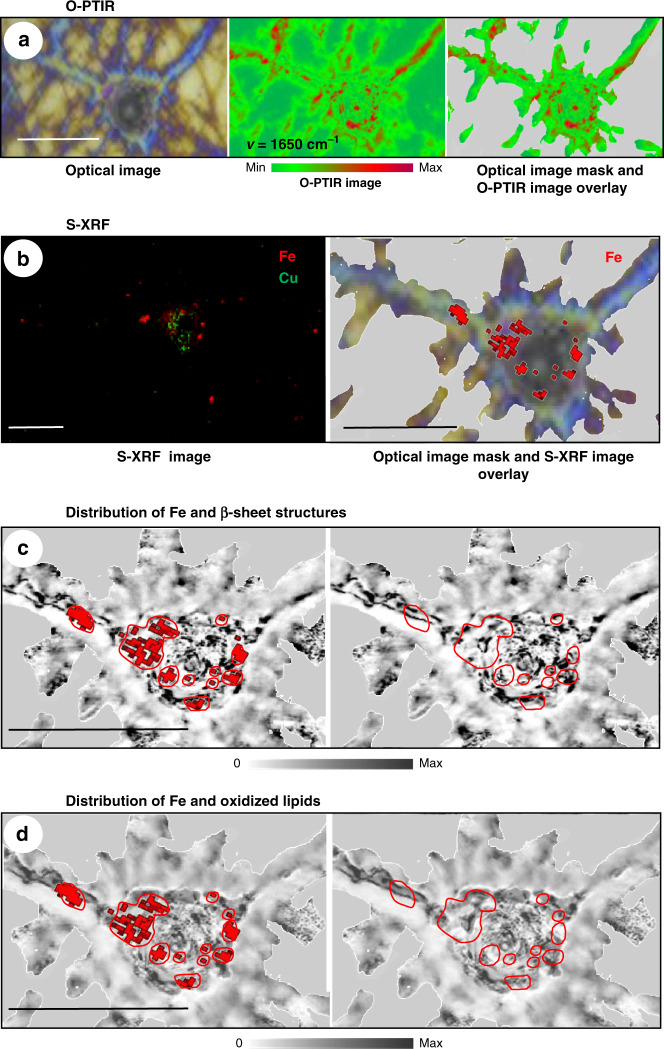
Correlative super-resolution optical photothermal infrared and scanning X-ray fluorescence imaging of individual neuron. **a** Left panel: Bright-field image of APP-KO primary neuron incubated with 5 × 10^−7^ M synthetic Aβ(1–42). Middle panel: O-PTIR maps acquired at frequencies 1650 cm^−1^. Color bar shows the intensity of photothermal amplitude. Right panel: overlay of a bright-field image with the mask created by an intensity threshold. **b** Left panel: Elemental distribution of Fe and Cu in the same neuron shown in (**a**). Right panel: overlay of Fe map with the masked visual image. **c** Left panel: Overlay of Fe map with the O-PTIR ratio map derived from the image acquired at 1628 cm^−1^ and divided by the image acquired at 1650 cm^–1^. Fe clusters are encircled. Distribution of β-sheet structures, encircled area indicate overlap with Fe. Red circles indicate overlap of Fe clusters and elevations in the β-sheet structural content. **d** Left panel: a ratio map was derived from the image of the same neuron shown in (**a**, **b**) acquired at 1740 cm^−1^ and divided by the image acquired at 1650 cm^−1^. Right panel: overlay oxidized lipid map with the mask. Red circles indicate overlap of Fe and overlay oxidized lipids. Scale bars are 20 µm

Thus, for single-cell integrative imaging, we used the match between the information depth (the full thickness of the neurons with IR and S-XRF were probed) and the ≈300 nm spatial resolution that allowed us to combine the two label-free nanospectroscopic techniques. Using the (x,y) coordinates that were first pre-selected based on the bright-field view, as shown schematically in Fig. S5, the same neurons were imaged by O-PTIR and S-XRF with a µm precision. Using O-PTIR, we visualized the distribution of β-sheet structures and oxidized lipids; then, using S-XRF, we visualized the distribution of metal ions in the same neurons. The spatial correlation of metal and structural maps allowed us to correlate the positions of iron clusters with elevated β-sheet structures and oxidized lipids (Fig. 4). Our results demonstrated a significant level of co-localization of clustered iron with elevated β-sheet structures, indicating that treatment with Aβ(1–42) causes Fe dyshomeostasis, which may cause lipid oxidation. To confirm that, mouse neuroblastoma N2aAPPswe cells (neuronal-like cells that overexpress Aβ) were treated with 10^−2^ M FeSO_4_ for 2 h. After treatment, we observed a significant increase in the intensity of the 1740 cm^−1^ band (Fig. S6). Moreover, by using S-XRF, we examined the brain tissue of AD transgenic mice and observed significant clustering of Fe (Fig. S7). To our knowledge, no such observation has been reported. Importantly, our results indicate that Fe but not Cu can be primarily involved in neurotoxicity in AD pathology. Thus, our results can partially explain a failure of clinical trials targeting Cu for AD treatment^6^.

**Fig. 5:**
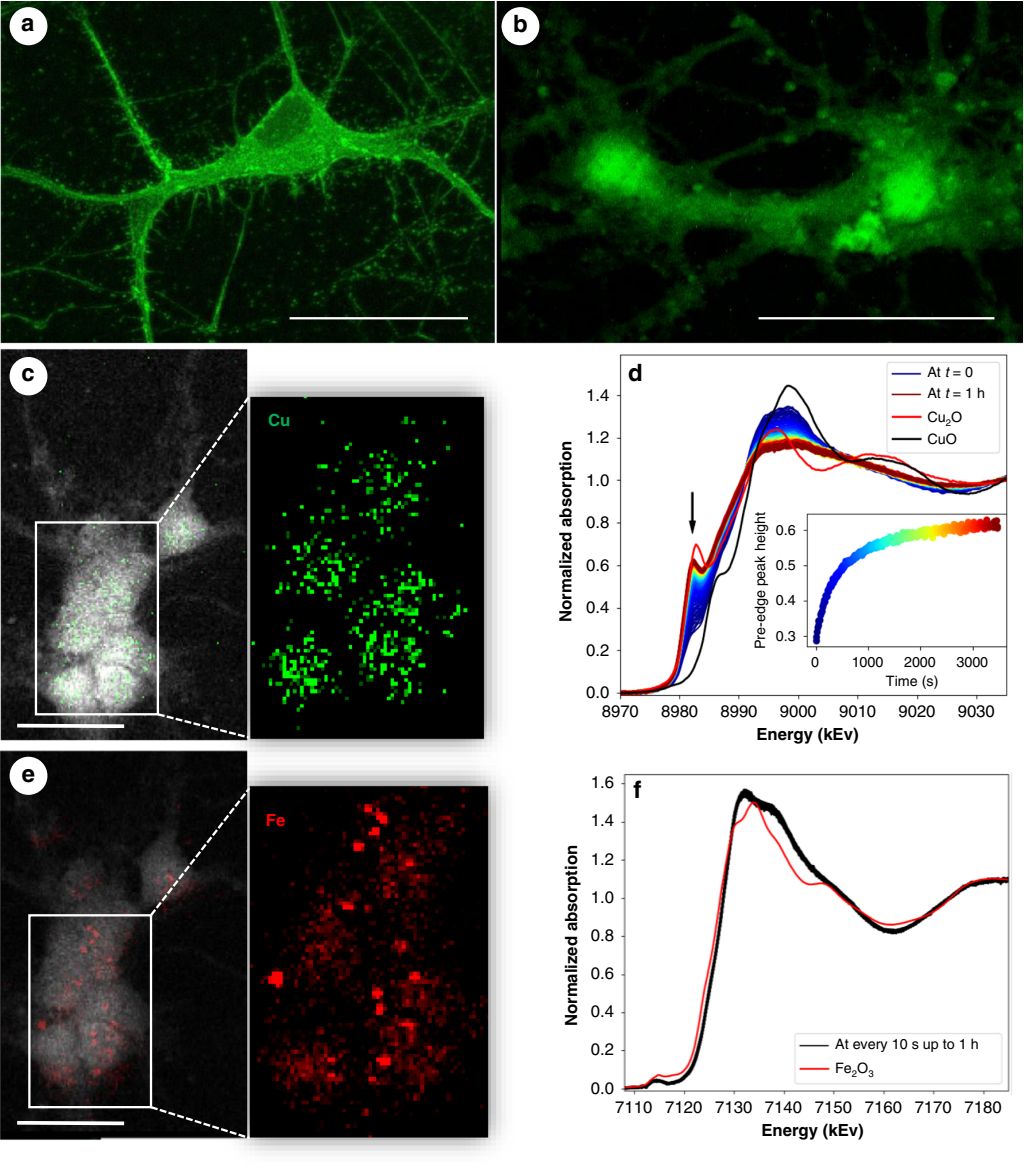
Immunofluorescence labeling of primary neurons after S-XRF experiment. **a** Representative immunofluorescence image of primary neurons labeled with neuron-specific antibody drebrin. **b** Drebrin immunofluorescence labeling of primary neurons after X-ray exposure. **c**, **e** Representative image of Cl, Cu, and Fe distributions in primary APP-KO neurons. **d** Photoreduction of copper during X-ray absorption measurements at room temperature. Cultured cells were incubated with 10^−2^ M CuSO_4;_ after a 30-min incubation, cells were washed, collected, and freeze-dried. XANES spectra were collected at room temperature. XANES spectra of Cu_2_O and CuO were used as references of Cu(I) and Cu(II), respectively. An arrow indicates the pre-edge peak at 8982.5 eV, indicative of the Cu(I) state. The inserted graph shows the dynamic of Cu photoreduction over the measurement time. **f** No photoreduction of iron was observed during X-ray absorption measurements at room temperature. Cultured cells were incubated with 10^−2^ M Fe_2_O_3;_ after a 30-min incubation at 4 °C, cells were washed, collected, and freeze-dried. XANES spectra were collected every 10 s for 1 h at room temperature

Here, we used label-free conditions; therefore, immunolabeling would be the last step of the study. However, after dehydration and X-ray exposure, none of the tested antibodies (Drebrin, Map2, 6E10, Table S2) were efficient. Our results indicated that sample dehydration and X-ray exposure may interfere with the subsequent immunolabeling (Fig. 5a, b). In addition, we tested whether hard X-ray exposure could cause photoreduction of Cu and Fe in the freeze-dried cells. For that purpose, we treated N2a cells with Cu_2_SO_4_ and FeO_3_ (Cu(I) and Fe(III) correspondingly). After treatment cells were collected and cell pellets were freeze-dried. X-ray absorption near edge structure (XANES) spectra were collected at the Balder beamline at the MAX IV synchrotron (Sweden). Our results demonstrated the pre-edge peak at 8982.5 eV, indicative of the Cu(I) state, and showed the dynamic of Cu(II) photoreduction over the measurement time (Fig. 5d). However, in the case of Fe(III), no photoreduction over the measurement time (spectra were collected every 10 s during 1-hour exposure) was detected (Fig. 5f) indicating that the current experimental setup can be used for further studies of the valence states of Fe. As for further Cu studies, photoreduction of Cu^2+^ under high X-ray flux must be considered, and cryo-conditions have to be implemented for the S-XRF measurements. The next step for further development of the approach is to improve the spatial resolution of O-PTIR by implementing a 405 nm wavelength probe laser instead of 532 nm, thus achieving 200 nm spatial resolution. Our approach could also be improved by developing protocols for a big data workflow and correlating larger datasets to provide sufficient sampling of cells required for statistical relevance.

## Discussion

In summary, here we presented a proof of concept of O-PTIR/S-XRF for single-cell imaging. We demonstrated that a combination of O-PTIR and S-XRF provides complementary information about structure and composition in heterogeneous biological samples such as neuronal cells providing more complete information about the system than if the two techniques were to be used separately. We analyzed co-localization of amyloid structural features and elemental distribution that can provide essential insights to the understanding of molecular reactions taking place at a subcellular level. Thus, using O-PTIR/S-XRF, we demonstrated a significant level of co-localization of clustered iron with elevated β-sheet structures, indicating that treatment with Aβ(1–42) may cause Fe dyshomeostasis, which in its turn, may cause lipid oxidation. To our knowledge, no such observation has been reported so far. Our results indicate that Fe but not Cu can be primarily involved in AD pathology.

Thus, we strongly believe that the O-PTIR/S-XRF imaging provides a motivation to use multimodal, interdisciplinary approaches for the studies that aim to understand neurodegeneration mechanisms related to amyloidopathy, such as AD.

## Materials and methods

### Sample preparation

APP knockout (APP-KO) neurons lack a functional APP gene; therefore, amyloid proteins, Aβ, and proteolytic cleavage products of APP are not expressed^22^. Consequently, Aβ instances containing β-sheet structures are not formed in these neuronal cells^4^. Primary neurons were cultured following the ethical guidelines and were approved by the Lund University Ethical Committee (M46-16). Primary neurons were isolated from APP-KO (Jackson Labs, Maine, U.S.A., JAX 004133) mouse embryos at embryonic day 16, as described in Martinsson et al.^22^. Neurons were seeded directly on a 5 × 5 mm, 100 nm-thick Si_3_N_4_ membrane on a 1 × 1 cm Si frame (Silson, UK). For culturing, Neurobasal medium with added glutamine, B27, and penicillin/streptomycin (Thermo Fisher Scientific, Sweden) was used. Before plating, Si_3_N_4_ membranes were coated with poly-d-lysine of molecular weight >300,000 (Sigma-Aldrich, Sweden), followed by rinses in Milli-Q water. Neurons were plated in 10% FBS and 1% penicillin-streptomycin in Dulbecco’s modified Eagle medium (DMEM; Thermo Fisher Scientific, Sweden); after 3 to 5 h; after cell adhesion, the media were exchanged for an FBS-free complete Neurobasal medium. One embryo was used for one set of cultures; experiments were reproduced 3–4 times. The following procedure was carried out to avoid artificial β-sheet formation: Neurons were fixed with 4% paraformaldehyde (Sigma-Aldrich, Sweden) in phosphate buffer saline for 15 min at room temperature, washed three times with MiliQ^R^, and freeze-dried. Specifically, water was removed from the well, and membranes were placed upright and stored at −80 °C until needed. Freeze-drying was selected over various dehydration processes for XRF sample preparation^41^, as more suitable to avoid lipid oxidation during air-drying and to preserve trace elements such as Cu and Fe. Examination of the sample using AFM confirmed the high quality of the sample preparation; a representative image is added in the supplementary information (Fig. S8).

### Optical photothermal infrared (O-PTIR)

O-PTIR imaging was performed at the SMIS beamline of the SOLEIL synchrotron (France). The detection scheme can be considered as a pump/probe experiment. The IR source was a pulsed, tunable four-stage QCL device, scanning from 1800 to 800 cm^−1^ at 80 kHz repetition rate. The probe was a CW 532 nm visible variable power laser, the photothermal effect was detected through the modulation of the green laser intensity induced by the pulsed IR laser. Further details about the fundamentals of the technique and the instrument itself can be found in references^16,20^ and in the Supplementary Information. Spectra were averaged for 20–50 scans with 1 s acquisition time per spectra to generate data of sufficient signal-to-noise ratio. Discrete frequency O-PTIR maps were collected at 500 nm step size. The collection parameters were: spectral range 1800–800 cm^−1^, reflection mode at 2 cm^−1^ spectral resolution, IR power set at 100% (< 0.6 mW), to avoid photodamage, the probe power was set to 5% (~2 mW). Background spectra were collected on an aluminized mylar background standard. Before analysis, intensity jumps arising around the transition wavelength between different QCL laser stages were removed using the PTIR Studio software. O-PTIR spectra were normalized and averaged; second-order derivation of the spectra was used to increase the number of discriminative features; the Savitsky−Golay algorithm with a 10-point filter with 3rd polynomial order was employed in this process. The β-aggregation level of proteins was visualized by calculating the map intensity ratio between 1628 cm^−1^, a peak corresponding to β-sheet structures, and 1650 cm^−1^, a peak corresponding to an α-helical structure. The increase of intensity in the resultant ratio map was considered a sign of the higher concentration of amyloid fibrils^24^ The intensity increase in the (1740 cm^−1^)/(1650 cm^−1^) ratio map was considered a sign of lipid oxidation^26,42^. To create masks, we used ImageJ^43^ and IrfanView (Copyright © 1996-2021 by Irfan Skiljan).

### Synchrotron-based X-ray fluorescence (S-XRF)

The S-XRF nano-imaging experiments were carried out at the Nanoscopium beamline^14^ at the SOLEIL synchrotron (France). The S-XRF elemental distributions were acquired using a 12.5 keV X-ray beam energy, which is above the K absorption edges of the elements of interest in this study (Cl, Fe, Cu, Zn). A Kirkpatrick–Baez nano-focusing mirror pair was used to create a high-intensity nano-beam with 10^10^ ph s^−1^ intensity. The flexible optical design and multi-length scale capability of Nanoscopium permitted the tuning of the spatial resolution to O-PTIR, namely 300 nm. The XRF spectra, one for each pixel, were measured with silicon drift diode detectors (SDD, Fig. 1). The S-XRF multi-elemental imaging technique enabled the simultaneous detection of the subcellular distribution of phosphorous, sulfur, potassium, calcium, iron, copper, and zinc. Elemental distributions were mapped using the Flyscan continuous sample scanning technique^44^. To map the trace element distribution in single neurons with high analytical sensitivity, we used 300 ms of dwell time per pixel. The subcellular elemental distribution maps were reconstructed online from the characteristic X-ray line intensities of the identified elements by a Matlab code created at Nanoscopium. For imaging, we selected individual neurons to avoid significant differences in sample thickness. The average size of clusters was calculated in the whole area of the cell, including axons and soma, using the software FIJI^43^.

Scanning X-ray fluorescence microscopy of the brain tissue samples was conducted at the NanoMAX hard X-ray nanoprobe beamline at the MAX IV synchrotron facility (Sweden). An X-ray beam with a photon energy of 12 keV was focused to a probe size of 200 nm. The brain tissue deposited on the Si_3_N_4_ membrane was raster scanned perpendicular to the focused beam. XRF spectra were recorded, one for each pixel, with a silicon drift diode detector. Each tissue scan was a rectangular area of 80 × 100 μm^2^ acquired with 0.2 s recording time per pixel and 200 nm pixel size. The photon flux incident on the sample was measured with an X-ray intensity monitor, simultaneously with the XRF signal. All measurements were done in air. XRF spectra for all samples were normalized to the photon intensity signal. XRF spectrum fitting, calibration to the XRF standard, and generation of elemental images for Cl, Cu, and Fe were done using the software PyMCA 4.5.4 (2004–2019 European Synchrotron Radiation Facility (ESRF)).

### Sample positioning

During O-PTIR microscopy, the (x, y) coordinates of the regions of interest were recorded relative to the corner of the Si_3_N_4_ membrane (Fig. S6). Using these coordinates, the area of interest was found with a micrometer precision at Nanoscopium using the optical microscope of the nanoprobe station and was refined by fast XRF mapping with micrometer spatial resolution. The precise location of the single neurons of interest was then chosen from the measured survey elemental maps for higher, 500 nm resolution and high elemental sensitivity mapping.

### X-ray absorption near edge structure (XANES)

The XANES spectra were measured at Balder beamline at MAX IV Laboratory (Lund, Sweden). Each scan took ~7 s, and the return motion of the Si <111> monochromator took ~3 s, making a 10 s spectrum-to-spectrum time difference. For these measurements, the monochromatic beam was focused to ~100 × 100 µm². For analysis, the background has been subtracted, and spectra were normalized.

### Confocal microscopy

Immunolabeling was done as recommended by the primary antibody manufacturer. Confocal images were obtained using a Leica TCS SP8 confocal microscope (Leica Microsystems) equipped with Diode 405/405 nm and Argon (405, 488, 552, and 638 nm) lasers with an HP PL APO 63x/NA1.2 water immersion objective.

## Supplementary information

Supplementary information

## Data Availability

The data that support the findings of this study are available from the corresponding author upon reasonable request.

## References

[1] Selkoe DJ, Hardy J (2016). The amyloid hypothesis of Alzheimer’s disease at 25 years. EMBO Mol. Med..

[2] Panza F (2019). A critical appraisal of amyloid-β-targeting therapies for Alzheimer disease. Nature Reviews. Neurology.

[3] Linse S (2020). Kinetic fingerprints differentiate the mechanisms of action of anti-Aβ antibodies. Nat. Struct. Mol. Biol..

[4] Klementieva O (2020). Super-resolution infrared imaging of polymorphic amyloid aggregates directly in neurons. Adv. Sci..

[5] Stephens AD, Zacharopoulou M, Schierle GSK (2019). The cellular environment affects monomeric α-synuclein structure. Trends Biochem. Sci..

[6] Esmieu C (2019). Copper-targeting approaches in Alzheimer’s disease: how to improve the fallouts obtained from in vitro studies. Inorg. Chem..

[7] Bourassa MW (2013). Elevated copper in the amyloid plaques and iron in the cortex are observed in mouse models of Alzheimer’s disease that exhibit neurodegeneration. Biomed. Spectrosc. Imaging.

[8] Telling ND (2017). Iron biochemistry is correlated with amyloid plaque morphology in an established mouse model of Alzheimer’s disease. Cell Chem. Biol..

[9] Cheignon C (2018). Oxidative stress and the amyloid beta peptide in Alzheimer’s disease. Redox Biol..

[10] Bousejra-ElGarah F (2011). Iron(II) binding to amyloid-β, the Alzheimer’s peptide. Inorg. Chem..

[11] Syme CD (2004). Copper binding to the Amyloid-β (Aβ) peptide associated with Alzheimer’s disease: folding, coordination geometry, pH dependence, stoichiometry, and affinity of Aβ-(1-28): insights from a range of complementary spectroscopic techniques. J. Biol. Chem..

[12] Rival T (2009). Fenton chemistry and oxidative stress mediate the toxicity of the β-amyloid peptide in a *Drosophila* model of Alzheimer’s disease. Eur. J. Neurosci..

[13] Nunomura A (2006). Involvement of oxidative stress in Alzheimer disease. J. Neuropathol. Exp. Neurol..

[14] Dear AJ (2020). Kinetic diversity of amyloid oligomers. Proc. Natl Acad. Sci. USA.

[15] Kreuzer M (2020). Lipids status and copper in a single astrocyte of the rat model for amyotrophic lateral sclerosis: correlative synchrotron-based X-ray and infrared imaging. J. Biophotonics.

[16] Zhang DL (2016). Depth-resolved mid-infrared photothermal imaging of living cells and organisms with submicrometer spatial resolution. Sci. Adv..

[17] Lima C (2021). Imaging isotopically labeled bacteria at the single-cell level using high-resolution optical infrared photothermal spectroscopy. Anal. Chem..

[18] Spadea A (2021). Analysis of fixed and live single cells using optical photothermal infrared with concomitant Raman spectroscopy. Anal. Chem..

[19] Wang AJ (2021). Resolving nanocomposite interfaces via simultaneous submicrometer optical-photothermal infrared-Raman microspectroscopy. Adv. Mater. Interfaces.

[20] Kansiz M (2020). Optical photothermal infrared microspectroscopy with simultaneous Raman – a new non-contact failure analysis technique for identification of <10 μm organic contamination in the hard drive and other electronics industries. Microsc. Today.

[21] Arosio P, Knowles TPJ, Linse S (2015). On the lag phase in amyloid fibril formation. Phys. Chem. Chem. Phys..

[22] Martinsson I (2019). APP depletion alters selective pre- and post-synaptic proteins. Mol. Cell. Neurosci..

[23] Barth A (2007). Infrared spectroscopy of proteins. BiochimicaBiophysica Acta (BBA)-Bioenerg..

[24] Cerf E (2009). Antiparallel β-sheet: a signature structure of the oligomeric amyloid β-peptide. Biochem. J..

[25] Dreissig I (2009). Quantification of brain lipids by FTIR spectroscopy and partial least squares regression. Spectrochimica Acta Part A: Mol. Biomol. Spectrosc..

[26] Petibois C, Déléris G (2004). Oxidative stress effects on erythrocytes determined by FT-IR spectrometry. Analyst.

[27] Wells K (1995). Neural membrane phospholipids in Alzheimer disease. Neurochem. Res..

[28] García-Morales V (2015). Membrane-derived phospholipids control synaptic neurotransmission and plasticity. PLoS Biol..

[29] Fuchs B, Bresler K, Schiller J (2011). Oxidative changes of lipids monitored by MALDI MS. Chem. Phys. Lipids.

[30] Oleszko A (2015). Application of FTIR-ATR spectroscopy to determine the extent of lipid peroxidation in plasma during haemodialysis. BioMed. Res. Int..

[31] Arrondo JLR, Goñi FM (1998). Infrared studies of protein-induced perturbation of lipids in lipoproteins and membranes. Chem. Phys. Lipids.

[32] Galeb HA (2012). The impact of single and double hydrogen bonds on crystallization and melting regimes of Ajwa and Barni lipids. Food Res. Int..

[33] Muik B (2007). Two-dimensional correlation spectroscopy and multivariate curve resolution for the study of lipid oxidation in edible oils monitored by FTIR and FT-Raman spectroscopy. Analytica Chim. Acta.

[34] Rohman A, Che Man YB (2011). Application of Fourier transform infrared (FT-IR) spectroscopy combined with chemometrics for authentication of cod-liver oil. Vibrational Spectrosc..

[35] Sánchez-Alonso I, Carmona P, Careche M (2012). Vibrational spectroscopic analysis of hake (*Merluccius merluccius* L.) lipids during frozen storage. Food Chem..

[36] Takahashi H, French SW, Wong PTT (1991). Alterations in hepatic lipids and proteins by chronic ethanol intake: a high-pressure Fourier transform infrared spectroscopic study on alcoholic liver disease in the rat. Alcohol. Clin. Exp. Res..

[37] Verity JE (2009). Tracking molecular interactions in membranes by simultaneous ATR-FTIR-AFM. Biophys. J..

[38] Somogyi A (2015). Optical design and multi-length-scale scanning spectro-microscopy possibilities at the Nanoscopium beamline of Synchrotron Soleil. J. Synchrotron Radiat..

[39] Das S (2019). Manganese mapping using a fluorescent Mn^2+^ sensor and nanosynchrotron X-ray fluorescence reveals the role of the Golgi apparatus as a manganese storage site. Inorg. Chem..

[40] Hostachy S (2018). Graftable SCoMPIs enable the labeling and X-ray fluorescence imaging of proteins. Chem. Sci..

[41] Jin QL (2017). Preserving elemental content in adherent mammalian cells for analysis by synchrotron-based x-ray fluorescence microscopy. J. Microsc..

[42] Benseny-Cases N (2014). Microspectroscopy (μFTIR) reveals Co-localization of lipid oxidation and amyloid plaques in human Alzheimer’s disease brains. Anal. Chem..

[43] Schindelin J (2012). Fiji: an open-source platform for biological-image analysis. Nat. Methods.

[44] Medjoubi K (2013). Development of fast, simultaneous and multi-technique scanning hard X-ray microscopy at Synchrotron Soleil. J. Synchrotron Radiat..

