# Supplementary material for: Correlative optical photothermal infrared and X-ray fluorescence for chemical imaging of trace elements and relevant molecular structures directly in neurons
Source: Light Sci Appl. 2021 Jul 22;10:151. doi: 10.1038/s41377-021-00590-x (PMC8298485; doi:10.1038/s41377-021-00590-x)
Supplement: Supplementary file 1 — Supplementary information [file 41377_2021_590_MOESM1_ESM.pdf]

Supplementary information

Correlative Optical Photothermal Infrared and X-ray fluorescence for

chemical imaging of trace elements and relevant molecular structures

directly in neurons

Nadja Gustavsson[a], Agnes Paulus[a,b], Isak Martinsson[c], Anders Engdahl[a], Kadda Medjoubi[d], Konstantin Klementiev[e], Andrea Somogyi[d], Tomas Deierborg [b], Ferenc Borondics[d], Gunnar K. Gouras[c], Oxana Klementieva[a,f]*

[a] Medical Microspectroscopy, Department of Experimental Medical Science, Lund University, 22180 Lund, Sweden
[b] Neuroinflammation, Department of Experimental Medical Science, Lund University, 22180 Lund, Sweden
[c] Experimental Dementia Research, Department of Experimental Medical Science, Lund University, 22180 Lund, Sweden
[d] Synchrotron SOLEIL, L'Orme des Merisiers, 91192 Gif Sur Yvette Cedex, France
[e] MAX IV Laboratory, 22100 Lund, Sweden
[f] Lund Institute for advanced Neutron and X-ray Science (LINXS), 223 70 Lund, Sweden

*CORRESPONDING AUTHOR:

E-mail address: oxana.klementieva@med.lu.se

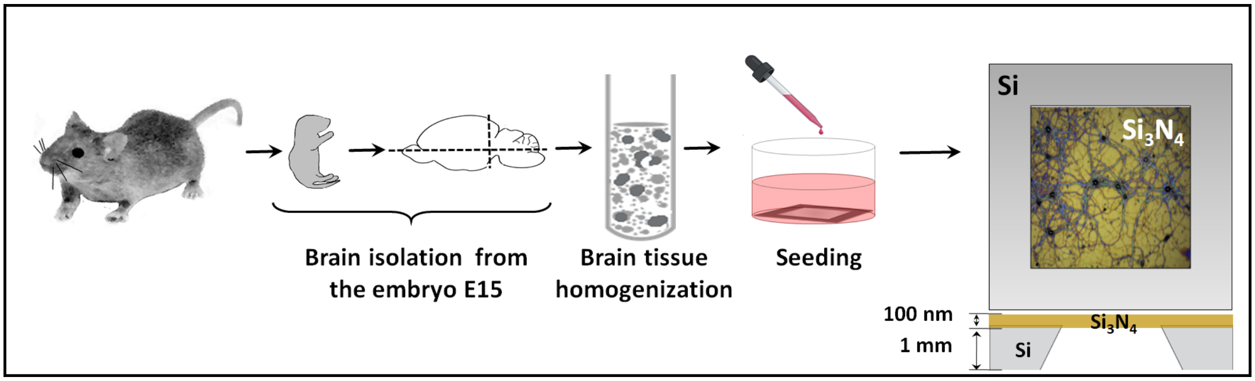

Figure S1. Sample preparation. Primary neurons isolation: cortex homogenate extracted from day 15 embryo was seeded on 100 nm Si_3N_4 membrane deposited on 1 mm thick Si. After 19 days of growth and maturation in culture, neurons were fixed, washed, and freeze-dried.

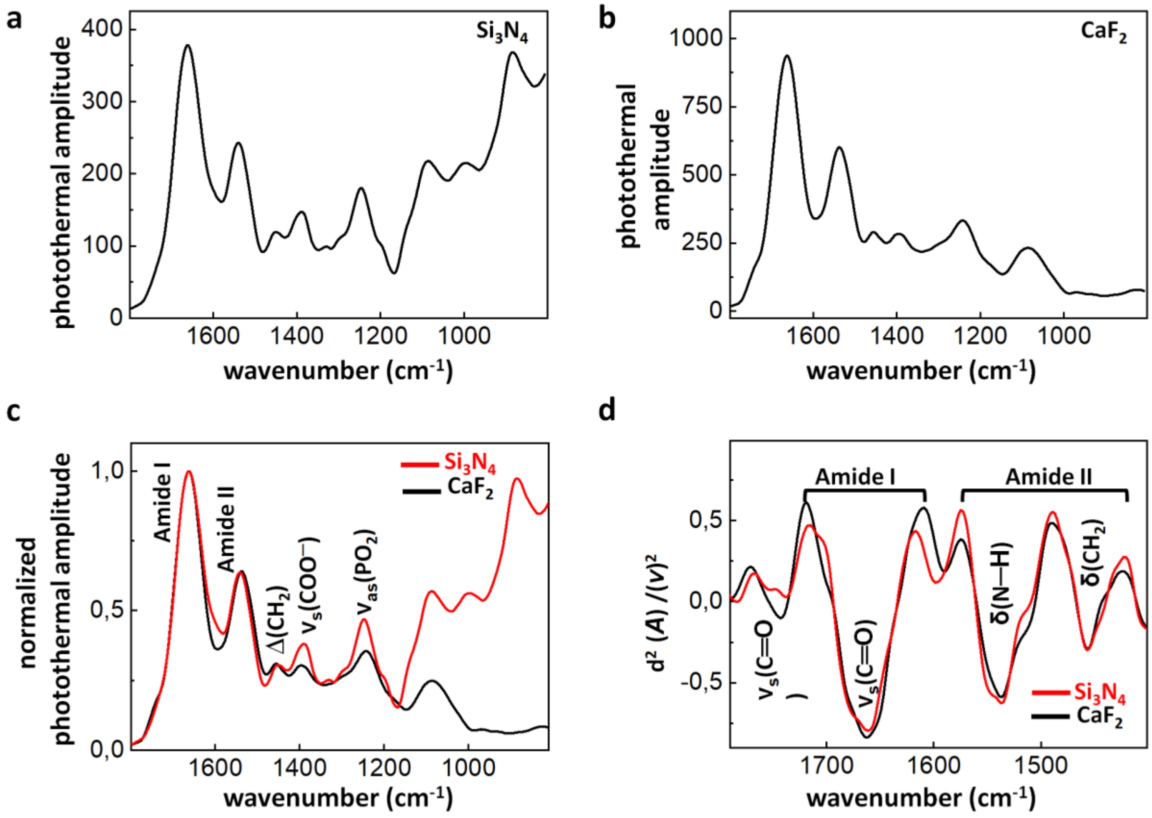

Figure S2. Substrate characterization. Averaged infrared spectra taken from primary neurons grown on Si_3N_4 (**a**) and CaF_2 (**b**). (**c**) An overlay of spectra shown in (a) and (b) with the indication of the main bands. The contribution of Si_3N_4 substrates is detected below 1200 cm^{-1} to the spectra. The observed difference in the intensities in 1400-1200 cm^{-1} may reflect metabolic variations in different neurons. (**d**) Averaged second derivatives showing the band positions used for the study. Spectra were acquired at 2 cm^{-1} spectral data point spacing with 50 averages, in reflection mode with the probe power set to 5% (~2 mW), the gain set to 50x. Background spectra were collected on an aluminized mylar background standard, IR 100% (<0.6 mW), probe power was set to 5% (~2 mW).

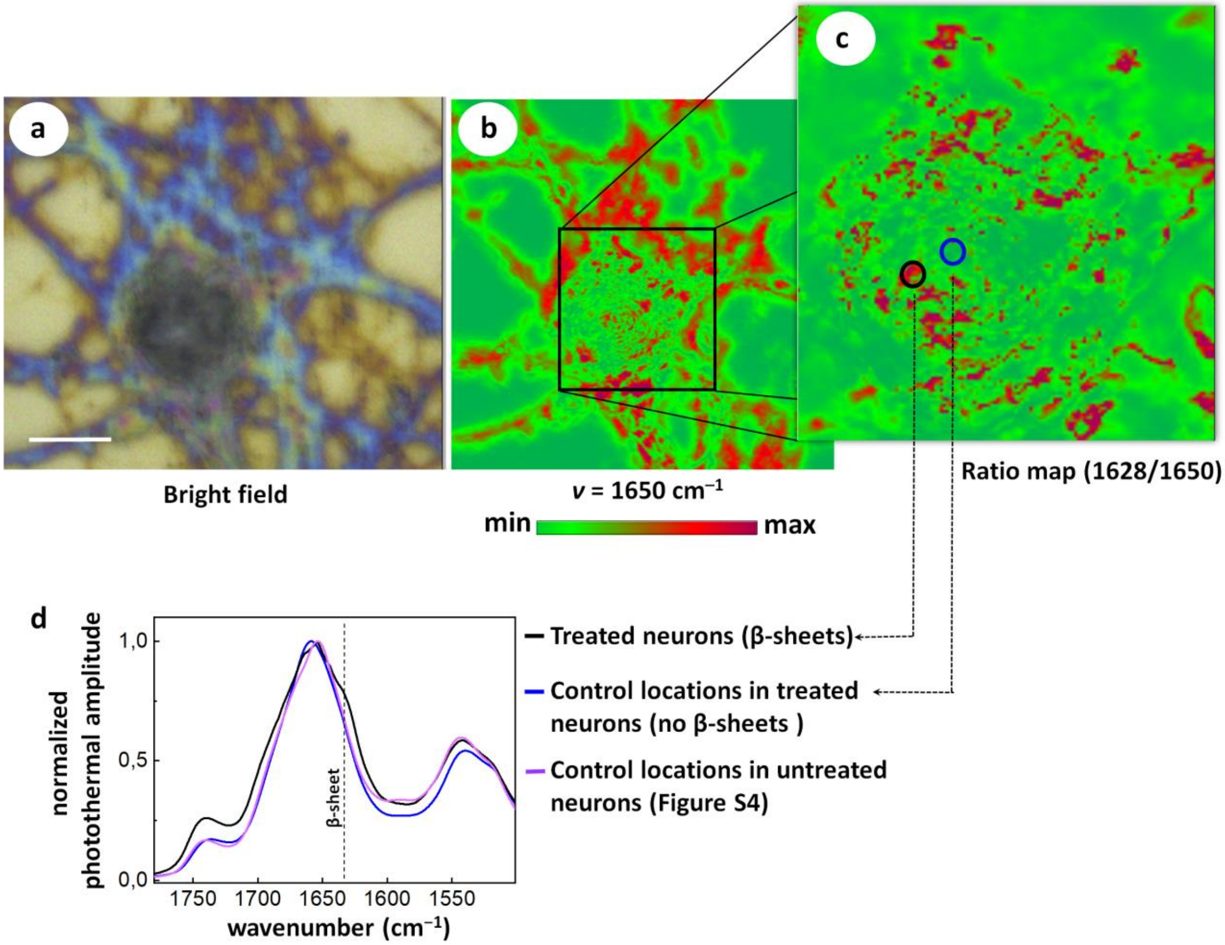

Figure S3. Spectra acquisition setup (**a**) Optical image of APP-KO neurons incubated 5×10^{-7} M synthetic Aβ(1–42) for 30 min. (**b**) O-PTIR map acquired at frequency 1650 cm^{-1}. (**c**) To select a location for a spectra acquisition, ratio maps (1628/1650) were calculated for treated and control neurons. Since a function to calculate map ratio is available in-build PTIR software, high precision was achieved in acquiring O-PTIR spectra from the calculated locations. In treated neurons, we observed elevations of β-sheet structures as red dots indicated by black circles, which were used for spectra acquisition. Blue circle indicates area without protein aggregation in treated neurons. (**a**) Averaged and normalized infrared spectra taken from the areas without protein aggregation in treated neurons, for black spectra a location shown by black circle in (d), for blue spectra a location shown by blue circle in (d); for magenta spectra taken from untreated neuron, corresponding locations are shown in Supplementary figures S4b and S4c, by magenta circles).

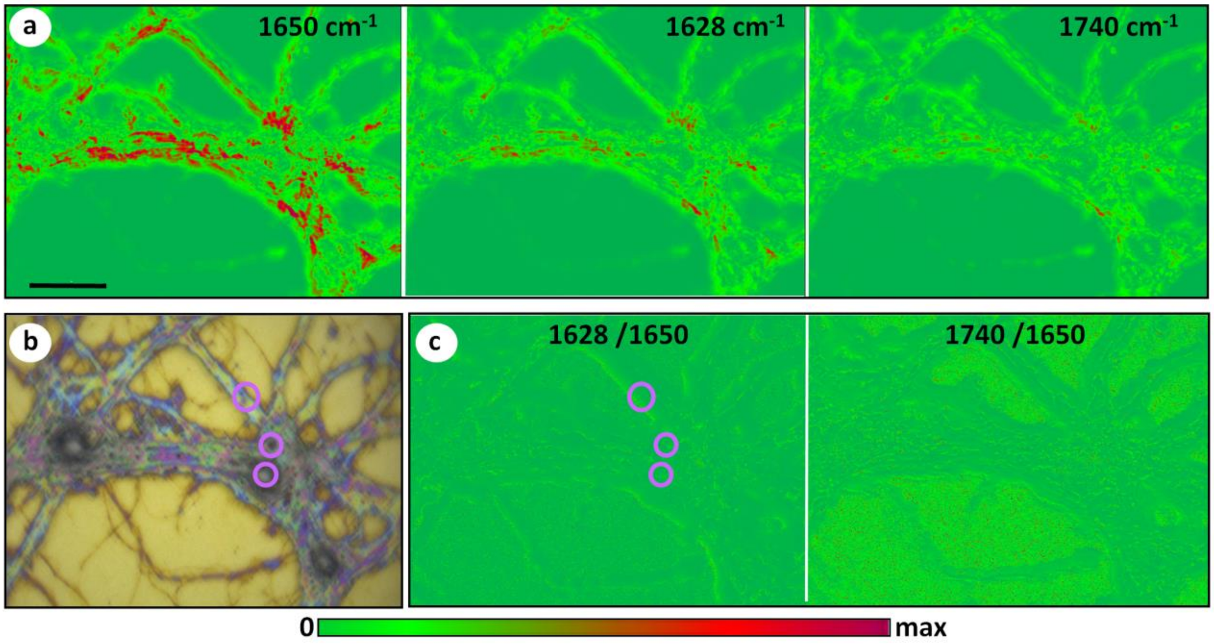

Figure S4. O-PTIR images of APP-KO primary neurons. A control sample, APP-KO primary neurons do not express APP and therefore lack Aβ that can not form β-sheet structures. (**a**) O-PTIR images acquired at the frequencies corresponding to 1650 cm^{-1}, 1628 cm^{-1} and to 1740 cm^{-1}. (**b**) Corresponding optical image of APP-KO neurons. (**c**) Ratio maps 1628/1650 and 1740/1650 show a lack of elevations of β-sheet structures and oxidized lipids. Magenta circles indicate example of randomly selected locations for spectra acquisition in healthy neurons, average spectrum is shown in Figure S3a. The colored scale shows the intensity of photothermal amplitude ranging from min (green) to max (red). Scale bar is 20 µm.

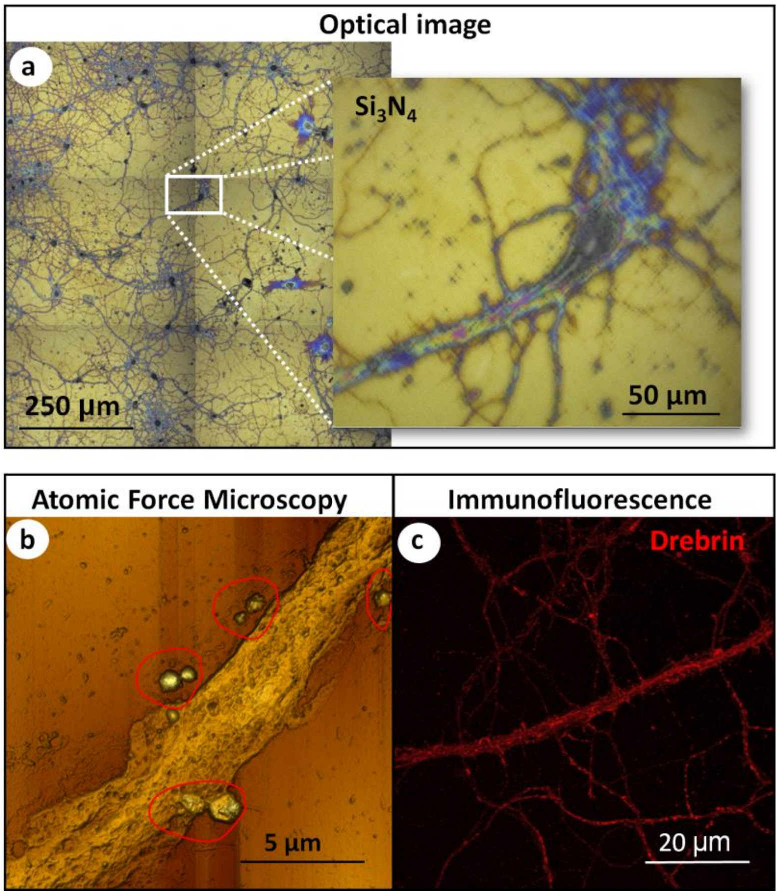

Figure S5. Sample preparation. (**a**) Primary neurons were seeded on the 100 nm thick Si_3N_4 membrane after 19 days of growth and maturation; neurons were fixed with 4 % paraformaldehyde in phosphate buffer saline for 15 min at room temperature, washed, and freeze-dried. Freeze-drying was selected over various dehydration processes for XRF sample preparation as more suitable to avoid lipid oxidation during air-drying and to preserve trace elements such as Cu and Fe. (**b**) Examining the neurons using AFM confirmed that freeze-drying does not affect the integrity of the neuronal membrane and preserves neuronal membranous protrusions and spines (encircled). (**c**) Confocal image showing the quality of immunolabeling with an antibody to Drebrin. Wild-type primary neurons were grown on glass, fixed with 4 % paraformaldehyde in PBS, washed with milli-Q water. The water was removed from the well, and coverslips were stored at -80 °C until needed. Before immunolabeling, neurons were rehydrated with 70-50-30-0 % ethanol in phosphate buffered saline.

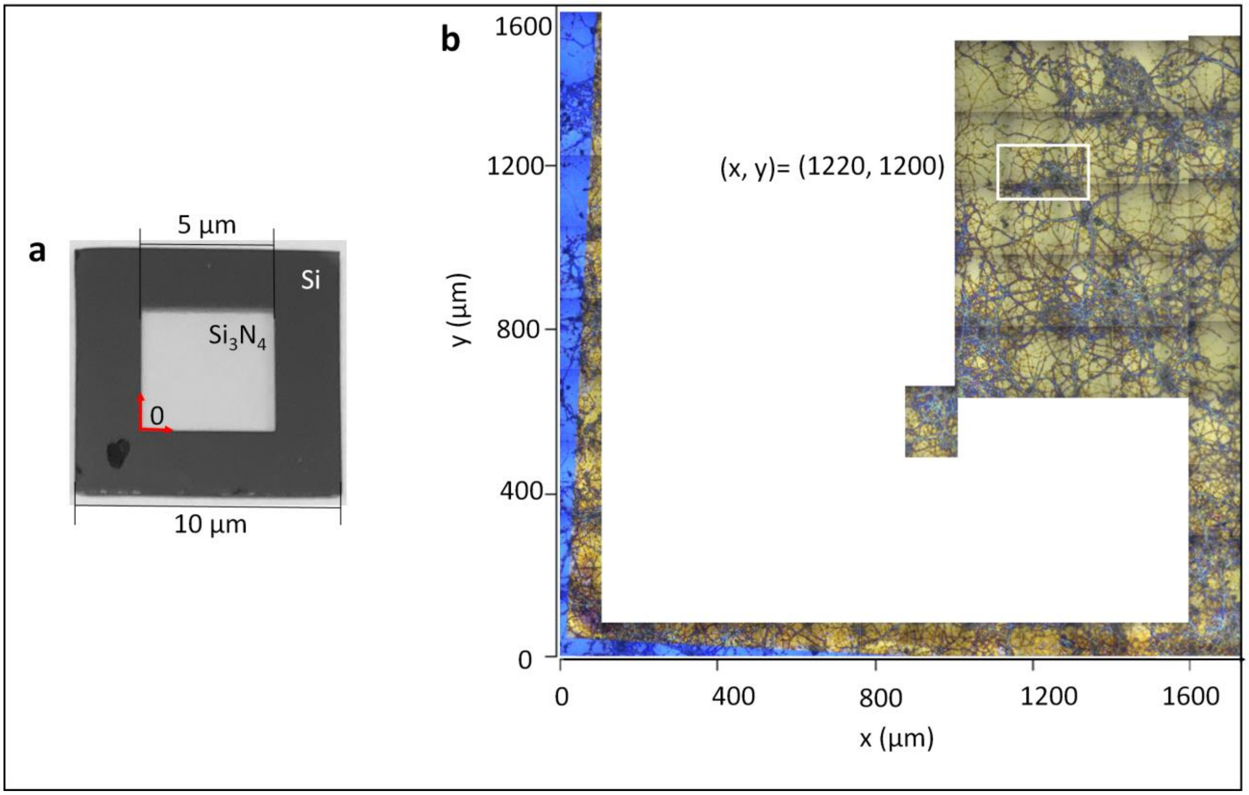

Figure S6. Sample positioning: **(a)** During O-PTIR microscopy, the (x,y) coordinates of the regions of interest were recorded relative to the corner of the Si_3N_4 membrane (red arrows) (**b**). For the study, we used 5 x 5 mm x 100 nm Si_3N_4 membrane on a 10 x 10 mm Si frame, square indicates a position for the measurements using two microscopes.

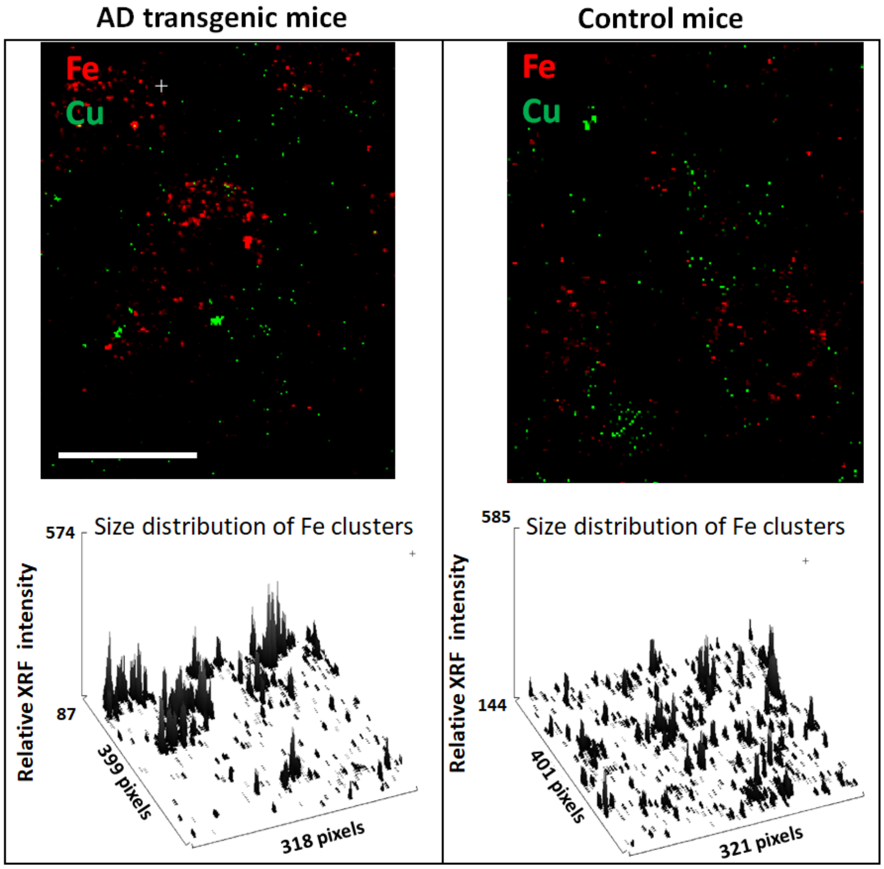

Figure S7. Elemental distribution of Fe and Cu in cryosectioned mouse brain tissue, transgenic AD model. All sections were 16 µm thick, and all images have the same area of 80 x 100 µm and were deposited on Si_3N_4. Pixel size was set to 200 nm; scale bar is 20 µm. The experiment was conducted at the NanoMAX hard X-ray nanoprobe beamline at the MAX IV synchrotron facility (Sweden). Left panel shows the presence of Fe clusters in the brain tissue of AD transgenic mice.

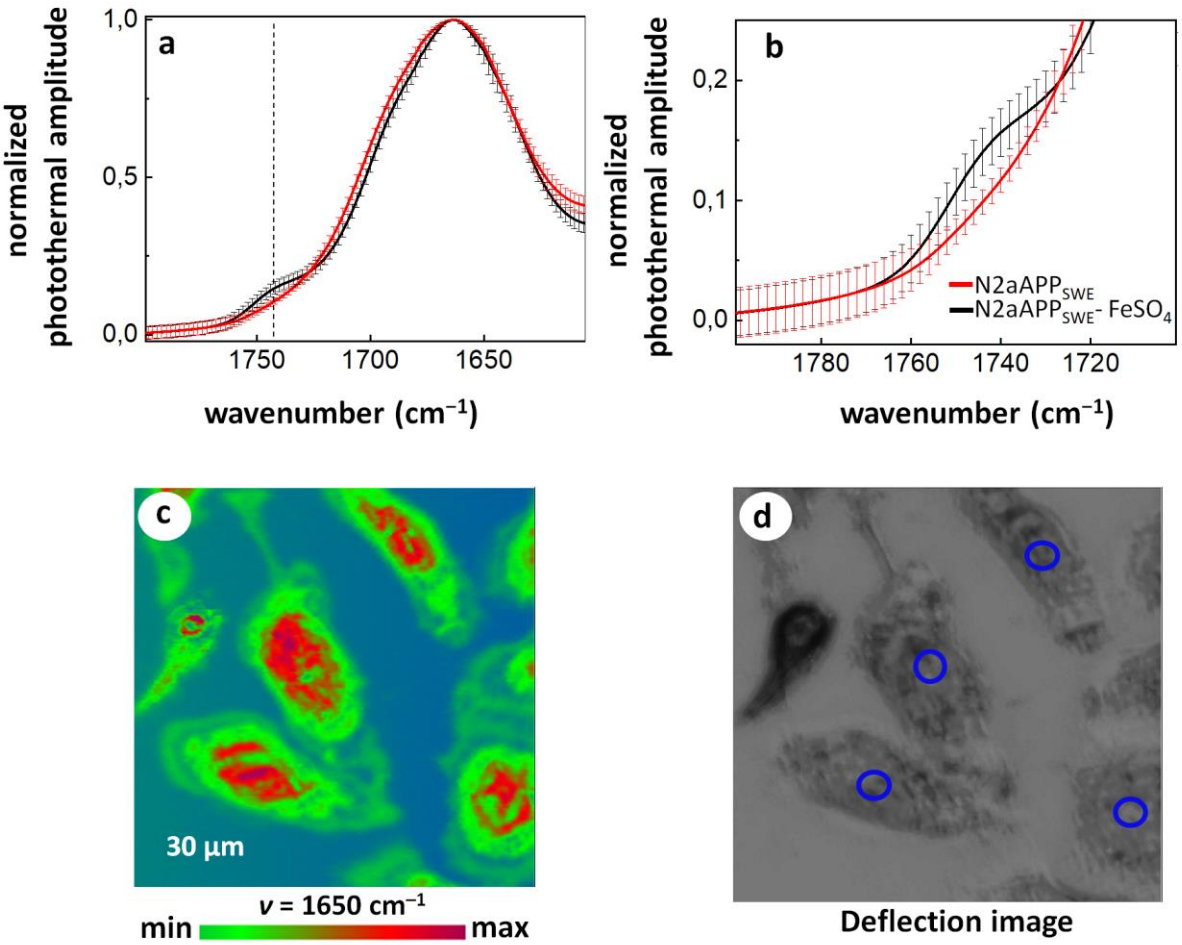

Figure S8. Effect of Fe on lipid oxidation. (**a, b**) Averaged infrared spectra taken from mouse neuroblastoma cells, N2aAPPswe. Red spectrum corresponds to N2aAPPswe cells endogenously overexpress amyloid-β protein. Black spectrum corresponds to the N2aAPPswe cells that were pretreated with 10^{-2} M $FeSO_4$ for 2 hours. (**c**) O-PTIR image of N2aAPPswe cells grown on Si_3N_4. (**d**) Corresponding deflection image showing O-PTIR spectra locations.

Wavenumber (cm^{-1})	Diffraction-limited resolution $\Delta x \geq 0.61(\lambda/(N\sin\Theta))$	
	**Objective 15x**	**Objective 32x**
4000	3.8 µm	2.3 µm
500	30.5 µm	18.8 µm

Table S1. Infrared diffraction limits, where NsinΘ is a numerical aperture.

Antibody	Target Epitope	Species and type	Dilution	Source	Cat. #
6E10	Human Aβ, full length APP, α/βAPP, α/β CTF, a.a. 3-8 of Aβ	Mouse monoclonal	1:500	BioLegend	Previously Covance catalog# SIG-39320
Drebrin	Drebrin	Rabbit polyclonal	1:1000	Abcam	ab11068
Map2	Map2	Chicken polyclonal	1:1500	Abcam	ab5392

Table S2. List of primary antibodies.
